# Bone metabolism is a key factor for clinical outcome of tibial plateau fractures

**DOI:** 10.1007/s00068-020-01537-4

**Published:** 2020-11-05

**Authors:** Matthias Krause, Lena Alm, Markus Berninger, Christoph Domnick, Kai Fehske, Karl-Heinz Frosch, Elmar Herbst, Alexander Korthaus, Michael Raschke, Reinhard Hoffmann, The “Fracture committee” of the German Knee Society

**Affiliations:** 1 Department of Trauma and Orthopaedic Surgery, University Medical Center Hamburg-Eppendorf, Martini Str. 52, 20246 Hamburg, Germany; 2 BG Trauma Hospital Hamburg, Hamburg, Germany; 3 Department of Trauma, Hand and Reconstructive Surgery, University Hospital Münster, Münster, Germany; 4 Department of Orthopaedic Trauma, Hand, Plastic and Reconstructive Surgery, University Hospital Würzburg, Würzburg, Germany; 5 Department of Trauma and Orthopaedic Surgery, Berufsgenossenschaftliche Unfallklinik Frankfurt Am Main, Frankfurt am Main, Germany

**Keywords:** Tibial plateau fracture, Bone metabolism, Ten-segment classification, Follow-up, Failure analysis

## Abstract

**Purpose:**

Given that tibial plateau fractures (TPF) are rare, they may pose a challenge to the treating surgeon due to their variety of complex fracture patterns. Numerous studies have identified potential fracture-specific, surgery-related, and patient-related risk factors for impaired patient outcomes. However, reports on the influence of bone metabolism on functional outcomes are missing.

**Methods:**

In a retrospective multicenter cohort study, 122 TPF of 121 patients were analyzed with respect to radiological and clinical outcomes (Rasmussen) with a mean follow-up of 35.7 ± 24.9 months. The risk factor assessment included bone metabolism-affecting comorbidities and medication.

**Results:**

The findings showed that 95.9% of the patients reported a good-to-excellent clinical outcome, and 97.4% reported a good-to-excellent radiological outcome. Logistic regression revealed that potentially impaired bone metabolism (IBM) was an independent risk factor for the clinical (*p* = 0.016) but not the radiological outcome (Table 4). Patients with 41-type B fractures and a potential IBM had a seven times higher risk to present a fair-to-poor clinical outcome [OR 7.45, 95 CI (4.30, 12.92)]. The most common objective impairment was a limited range of motion in 16.4% of the patients, especially in 41-type C fractures (*p* = 0.06). The individual failure analysis additionally identified surgery-related options for improvement.

**Conclusion:**

This study demonstrated that potential IBM was an independent risk factor for a poor-to-fair clinical outcome.

## Introduction

Given that tibial plateau fractures (TPF) are rare, they may pose a challenge to the treating surgeon due to their variety of complex fracture patterns [1]. There are numerous approach concepts to improve surgical outcome of TPF, including the updated three-column, the revised three-column, which is a mechanism-driven approach, and the ten-segment concept [1–5]. Nonetheless, persistent pain, impaired range of motion (ROM), and/or instability are repeatedly described. While short- and mid-term outcomes have been associated with a low return-to-sports rate and a low patient-related outcome compared to healthy individuals (Knee Injury and Osteoarthritis Outcome Score), a good-to-excellent knee function in the long term has been reported (Rasmussen and Hospital for Special Surgery Knee Scoring System) [6–10]. Several studies have identified potential fracture-specific and surgery-related risk factors for impaired patient outcomes [6–11]. As a result, there is general consensus that anatomical reconstruction within a 2–3-mm articular step-off, a straight leg axis, and stable bony and ligamentous fixation should be considered [9, 11–14].

While some studies have also analyzed the influence of patient-specific risk factors, medication, or comorbidities, their association with bone metabolism and its impact on patient outcomes has not been in the focus [6, 7, 10, 15–17]. Instead, impaired bone metabolism (IBM) and its association with patient-specific risk factors such as vitamin D deficiency, thyroid dysfunction, chronic kidney disease (CKD) or liver failure, systemic immunodeficiency after organ transplantation or with HIV, or osteoporosis itself have most commonly been studied in relation to fracture risk of vertebral and hip fractures. The influence of vitamin D deficiency on bone health is undisputed and seems to have a negative impact on fracture healing [18]. Other endocrine diseases like hyperthyroidism, renal, or liver failure are also common in descending order of prevalence and relate to increased fracture risk (hyperthyroidism: from 17 to 97%) depending on location of the fracture site [19, 20].

While the exact prevalence of IBM is unclear, many of its associated risk factors such as a vitamin D deficiency (up to 40.4%), which may be called pandemic in western Europe, CKD (up to 10.6%), and hyperthyroidism (up to 1.3%) are common [21–23]. Osteoporosis, the most obvious reason for IBM, is prevalent in 35% of all postmenopausal white women, further increasing with age [24]. Therefore, the impact of a potential IBM on fracture risk, subsequent fracture healing, and postoperative patient outcome in TPF is conceivable and still underrated.

The aim of this study was to analyze patient outcome after surgical treatment of TPF and to evaluate the potential influence of patient comorbidities affecting bone metabolism on surgical outcome. We hypothesized that patients with accompanying bone metabolism-affecting comorbidities or medication showed a worse outcome than patients without known potentially impaired bone metabolism (IBM). We also aimed to present an individual failure analysis of patients with a poor-to-fair functional outcome (Rasmussen) after open reduction and internal fixation (ORIF) of TPF.

## Patients and methods

### Study group

This retrospective multicenter cohort study analyzed all consecutive patients who were surgically treated at a level-one trauma center from June 2010 to December 2015 from a study group that was previously reported on [1]. We included all patients with a minimum follow-up of 12 months and excluded patients without a preoperative computed tomography (CT) scan, extra-articular fracture manifestation (Orthopedic Trauma Association/AO Foundation (OTA/AO) type A), and periprosthetic fractures. In addition to the assessment of patient-related outcome measures (Rasmussen), electronic data files were carefully searched for comorbidities that could potentially influence bone metabolism according to the present S3 guideline on the treatment of osteoporosis in Germany [25]. Hence, a potentially impaired bone metabolism (IBM) was defined as the presence of hyperthyroidism, primary hyperparathyroidism, Cushing syndrome, chronic kidney disease grade 3a and above, human immunodeficiency virus (HIV), diabetes mellitus, vitamin D deficiency (serum level < 50 nmol/L), rheumatoid arthritis, and/or osteoporosis according to the WHO standard definition of a femoral or hip DXA T-score -2.5 SD or lower. Specific risk factors, such as smoking, alcohol abuse, low body mass index (BMI), or medication, including chemotherapeutic agents, cortisol, and specific anti-retroviral medication were also assessed but not automatically categorized to an IBM. The study protocol was approved by the local ethics committee (WF-02/16).

### Surgical treatment

TPF were categorized according to the OTA/AO and 10-segment classification [1, 26–28]. All cases were surgically managed, including the choice of the surgical approach and potential placement of the osteosynthesis, based on the concept of the ten-segment classification to evaluate the intraarticular fracture run in addition to the 3-column concept for extra-articular fracture manifestation [27–31]. Management included the application of an external fixator in cases of serious soft-tissue damage and screw fixation in minor depression fractures (OTA/AO Type B2). Unilateral split-depression fractures were usually treated with screw and plate fixation (OTA/AO type B3), while bilateral fractures were treated with dual plate and screw fixation (OTA/AO type C). The number and combination of surgical approaches are given in Table 1. Certified trauma surgeons performed all procedures. Postoperatively, rehabilitation protocols differed, depending on the fracture pattern, soft-tissue injury, and surgical treatment. In general, all patients started with physiotherapy within the first week after soft-tissue consolidation. Except for some cases with ligament injuries or posterior fragment involvement, patients were mobilized without a knee brace and were recommended to gradually increase knee flexion. All patients were kept partial weight bearing for at least 6 weeks postoperatively.

**Table 1:** Patient characteristics with regards to fracture type according to OTA/AO classification (*n* = 122)

Characteristics	OTA/AO type B (*n* = 85)	OTA/AO type C (*n* = 37)	Type B vs. type C *p* value
Sex ratio (*n*; **♀/♂**)	48/37	19/18	Ns
Age (± SD, years; **♀/♂**)	52.5 ± 19.1/43.8 ± 12.7	60.1 ± 6.4/48.5 ± 8.4	0.014/ < 0.001
Mechanism of trauma			
High energy (*n*; **♀/♂**)	25/34	12/16	< 0.001/0.042
Low energy (*n*; **♀/♂**)	23/3	7/2	
Risk factors (*n*; **♀/♂**)			
Smoking	1/12	1/7	< 0.001/0.013
Diabetes	1/1	1/1	ns/ns
Impaired bone metabolism	10/4	5/5	ns/ns
BMI (mean: kg/m^2^)	22.0 ± 2.7/27.1 ± 5.2	27.0 ± 6.7/27.1 ± 2.8	< 0.001/ns
Surgical approaches (*n*)			
CRIF with jail technique	13	0	
Anterolateral	57	8	
Posterolateral	2	3	
Anteromedial	6	1	
Posteromedial	1	2	
Median	1	1	
Combination AL + PM	3	9	
Combination AM + PM	0	2	
Combination AM + AL	0	5	
Combination PL + PM	2	2	
Combination AL + AM + PM	0	3	
Combination AL + AM + PL	0	1	

*ns* not significant, *SD* standard deviation, *BMI* body mass index, *CRIF* closed reduction internal fixation, *AL* anterolateral, *PL* posterolateral, *AM* anteromedial, *PM* posteromedial

### Outcome measures

Patient-reported outcome measures and radiological follow-ups were obtained according to the clinical and radiological Rasmussen score [32]. Clinical and radiological outcome were categorized according to the total sum of Rasmussen score: clinical score—excellent (30–27 points), good (26–20), fair (19–10), poor (9–6); radiological score excellent (18 points), good (17–12), fair (11–6), poor (5–0) [33]. All patients were given a questionnaire regarding their subjective outcome and were evaluated with respect to their objective clinical findings. Radiological evaluation included preoperative and postoperative conventional X-rays in antero-posterior and lateral views at the time of their follow-up. Coronal alignment of the proximal tibia was assessed by analyzing the mechanic medial proximal tibial angle (norm 87 ± 3°) [12, 34]. Postoperative radiological follow-up was available for 76 patients. Evaluation of all radiological films was performed by an experienced consultant who specialized in orthopedic trauma.

### Statistical analysis

Statistical analysis was carried out using IBM^®^ SPSS^®^ Statistics 26 (SPSS Inc, Chicago, IL, USA). Data were presented as mean values ± standard deviation for continuous variables. A Student’s *t* test was used to determine the significance of differences between fracture groups, within group differences, and clinical and radiological categories and between the sexes. Differences among categorical data were tested with a Chi-square test. Binary logistic regression analysis was performed to test the influence of fracture type and the presence of a potential IBM on clinical and radiological outcomes (Rasmussen). All tests were two-sided, and a *p* value of < 0.05 was considered statistically significant.

## Results

### Study group

We included a total of 121 patients (males *n* = 55, females *n* = 66) with 122 TPF; one patient presented with bilateral TPF. The mean age at operation was 50.6 ± 15.4 years. The clinical follow-up period ranged from 12 to 111 months, with a mean of 35.7 ± 24.9 months. The radiological follow-up was significantly shorter (22.4 ± 12.5 months, *p* < 0.001). The baseline characteristics are presented in Table 1. Using the OTA/AO classification, we analyzed 85 cases with a 41-type B fracture [B1: *n* = 3 (2.3%), B2: *n* = 45 (35.2%), B3: *n* = 37 (28.9%)] and 37 cases with a 41-type C fracture [C1: *n* = 9 (7.3%), C2: *n* = 2 (1.6%), C3: *n* = 26 (21.3%)]. Two patients presented with an open fracture, and one compartment syndrome had to be treated. Twelve patients (9.8%) required temporary immobilization with an external fixator. The mean delay to final surgery due to soft-tissue consolidation was 4.1 ± 3.5 days (range: 0–25 days).

### Clinical and radiological outcome

Overall, 95.9% (*n* = 117) of the sampled patients reported a good-to-excellent clinical outcome. The radiological outcome was excellent in 76.3% and good in another 21.1% of the cases. Patients with 41-type B fractures showed a significantly better clinical (27.9 ± 2.8 vs. 26.0 ± 4.1; *p* = 0.015) and radiological (17.6 ± 1.3 vs. 15.8 ± 3.3; *p* = 0.021) Rasmussen Score than those with 41-type C fractures (Tables 2, 3, and 4). Inferior clinical knee function was not associated with the follow-up period. The most common objective impairment was limited ROM in 16.4% (*n* = 21) of the patients, especially in 41-type C fractures (*p* = 0.06).

**Table 2:** Mean clinical results to fracture type according to OTA/AO classification (*n* = 122)

	OTA/AO type B (*n* = 85)	OTA/AO type C (*n* = 37)	Type B/type C *p* value
Rasmussen (total)	27.9 ± 2.8	26.0 ± 4.1	0.015
Subjective			
Pain	5.1 ± 1.4	4.8 ± 1.6	ns
Walking capacity	5.5 ± 1.1	4.8 ± 1.6	0.018
Objective			
Extension	5.8 ± 0.7	5.5 ± 1.0	ns
Total range of motion	5.7 ± 0.8	5.2 ± 0.8	0.006
Stability	5.9 ± 0.4	5.8 ± 0.5	ns

*ns* not significant

**Table 3:** Mean radiological results according to OTA/AO classification (*n* = 122)

	OTA/AO type B (*n* = 54)	OTA/AO type C (*n* = 22)	Type B/type C *p* value
Rasmussen (total)	17.6 ± 1.3	15.8 ± 3.3	0.001
Depth	5.9 ± 0.5	5.0 ± 1.7	0.001
Wide	5.9 ± 0.6	5.8 ± 0.6	ns
Angulation	5.8. + 0.7	5.0. ± 1.3	0.001

*ns* not significant

**Table 4:** Clinical (*n* = 122) and radiological outcome (*n* = 76) according to categories: poor/fair vs. good/excellent

	Radiological poor/fair	Radiological good/excellent	Clinical poor/fair	Clinical good/excellent
Radiological poor/fair	–	–	1	1
Radiological good/excellent	–	–	2	72^#^
Clinical score				
Subjective				
Pain	2.0 ± 2.8	5.0 ± 1.3*	0.3 ± 0.8	5.2 ± 1.1*
Walking capacity	1.0 ± 1.4	5.5 ± 0.9*	1.7 ± 1.4	5.5 ± 1.0*
Objective				
Extension	5.0 ± 1.4	5.5 ± 0.9	5.2 ± 1.0	5.2 ± 0.8
Total range of motion	4.0 ± 0.0	5.6 ± 0.9*	4.7 ± 1.0	5.5 ± 0.8*
Stability	6.0 ± 0.0	6.0 ± 0.2	5.8 ± 0.4	5.9 ± 0.4
Radiological score				
Depth	1.0 ± 1.4	5.8 ± 0.7*	2.7 ± 1.2	5.8 ± 0.9*
Wide	5.0 ± 1.4	5.9 ± 0.5*	5.3 ± 1.2	5.9 ± 0.5
Angulation	3.0 ± 1.4	5.6 ± 0.9*	4.0 ± 0.0	5.6 ± 0.9*
Potential IBM (yes/no)	1/1	13/61	4/2	20/96^#^
Fracture category (Type B/C)	0/2	54/20^#^	3/3	82/34

*p* < 0.05

*Student’s *t* test

^#^Chi-square test: *p* < 0.05

### Influence of potential IBM

A potential IBM was detected in 19.7% of the patients (*n* = 24). These included osteoporosis (*n* = 7), hyperthyroidism (*n* = 6), high-grade liver cirrhosis (*n* = 3), severe vitamin D deficiency (*n* = 2, [35]), high-grade (> grade 3) chronic renal failure (*n* = 2), chronic type-c gastritis (*n* = 2, one patient with unspecified collagenosis), and HIV (*n* = 2). Logistic regression revealed that potential IBM was an independent risk factor for the clinical (*p* = 0.016) but not for the radiological outcome (Table 5). Patients with 41-type B fractures and potential IBM had a seven times more likely risk of having a fair-to-poor clinical outcome [OR 7.45, 95 CI (4.30, 12.92)].

**Table 5:** Logistic regression with respect to clinical outcome (Rasmussen: poor and fair/good and excellent)

Variable	B	SE	Wald	df	Sig	Exp (B)
Model	3.461	0.885	15.302	1	< 0.001	31.847
Metabolic bone disorder	− 2.182	0.908	5.774	1	0.016	0.113
Fracture type (41-type B or C)	0.621	0.879	0.499	1	0.480	1.861

Mean BMI was 25.2 ± 4.9 kg/m^2^. Only three patients presented with an underweight BMI (< 18.5 kg/m^2^). Smoking was observed in 17.2% of the patients (*n* = 21). Smoking, BMI, and diabetes showed no significant association with either the radiological or the clinical outcome measures.

### Individual failure analysis

**Fig. 1:**
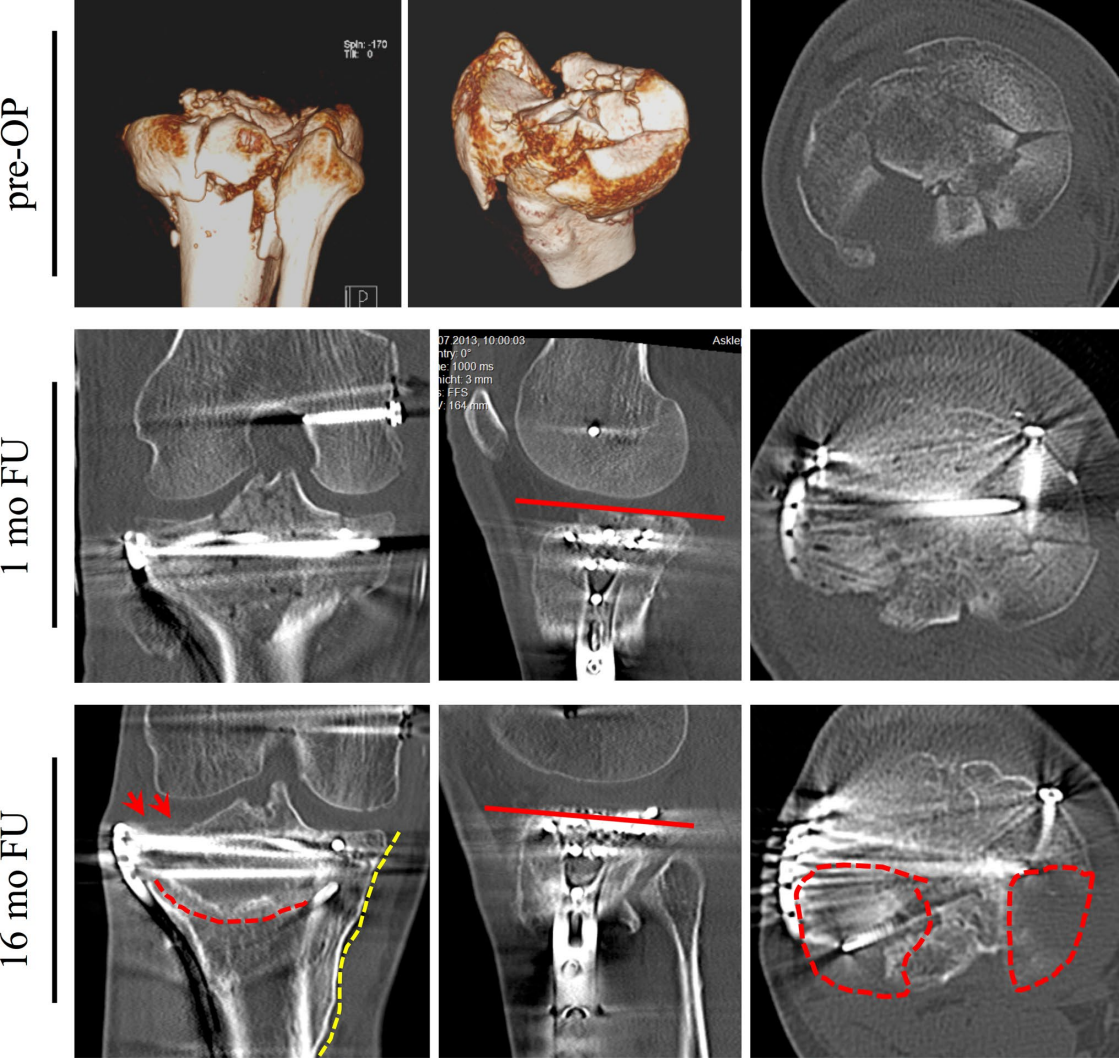
A 50-year-old male with a bicondylar TPF [AO 41-type C3, medial split, lateral comminution involving the tibial spine and the most severe depression in the postero-latero-central (PLC) segment, *pre-OP*] after a motorcycle accident. A 1-month follow-up (FU) after lateral unilateral plating and antero-posterior screw fixation of the medial plateau showed anatomic articular reconstruction medially and laterally via an anterolateral and posteromedial approach, including an osteotomy of the medial femoral epicondyle for improved visualization (*1-month FU*). At the 16-month FU, lateral and medial loss of reduction (red arrows in anterior–posterior (ap) view and red line in sagittal view) with a substantial subchondral bone defect (red dotted line in ap view) accompanied with a medial bony union (yellow dotted line) could be observed. Primarily, the postero-latero-lateral and the PLC segments were affected (red dotted line in axial view). The patient presented with bone metabolism-affecting comorbidities, including human immunodeficiency virus with anti-retroviral therapy and a long-term proton-pump inhibitor prescription

**Fig. 2:**
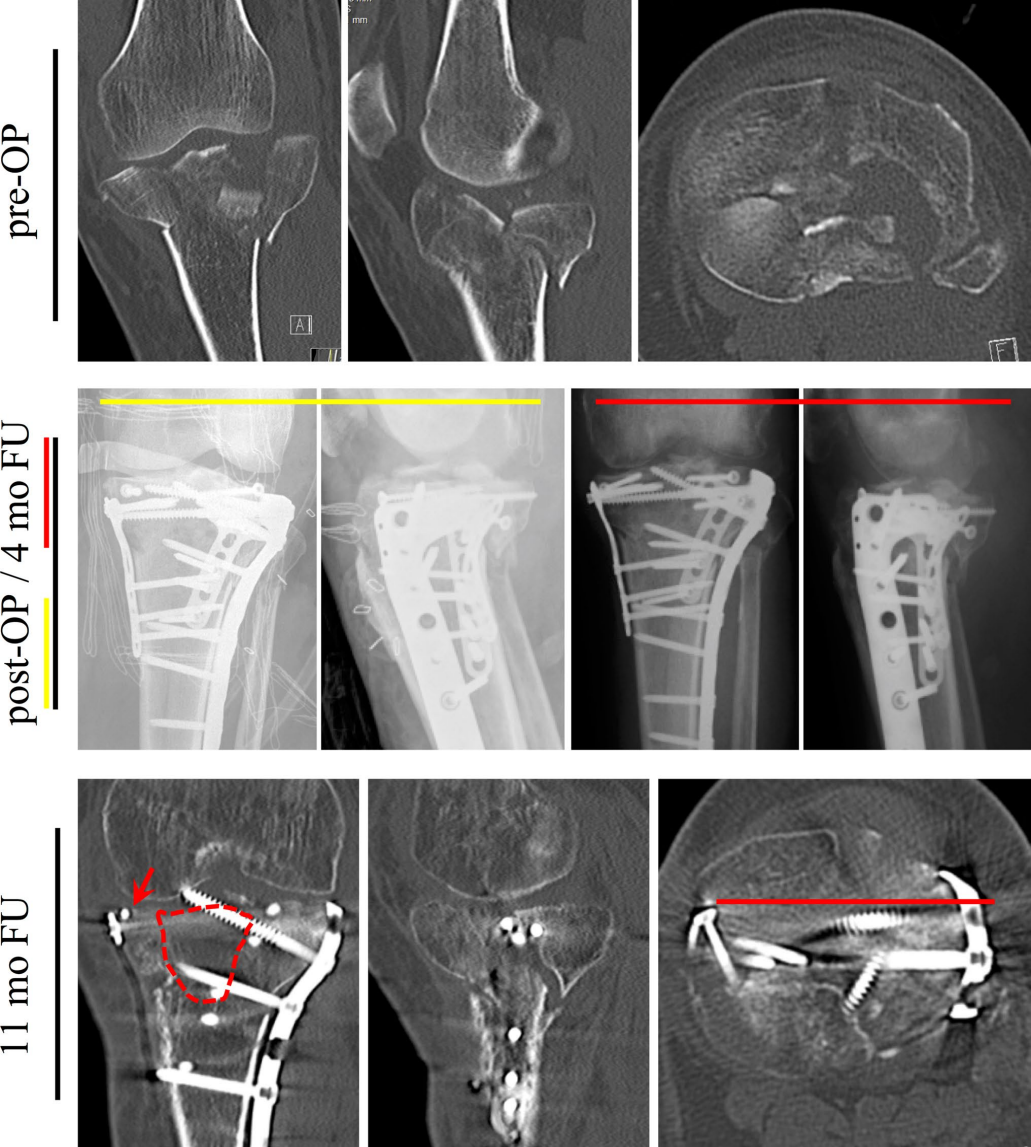
A 50-year-old male with a bicondylar TPF (AO 41-type C3, medial split, lateral comminution involving the tibial spine and the most severe depression in the postero-latero-central segment, *pre-OP*) after a fall due to alcohol intoxication. A postoperative X-ray of the lateral and posterolateral reduction with individual plate fixation in addition to anatomic reduction and medial plate fixation of the medial tibial plateau (yellow head line). Medially, an additional jail screw supported subchondral fixation (*post-OP)*. The 4-month follow-up (FU) showed secondary loss of reduction (red arrow in the anterior–posterior view) with a substantial subchondral bone defect (red dotted line), secondary intraarticular screw location, and an osteopenic bone stock (*4-mo FU*). The sagittal and axial views revealed an unfortunate screw placement into the fracture gap without providing sufficient stabilization. While the antero-posterior jail screw might not have had enough osteosynthetic subchondral bone support, there was no posteromedial buttress. Comorbidities included chronic hypocalcemia, chronic alcohol abuse, and long-term smoking

**Fig. 3:**
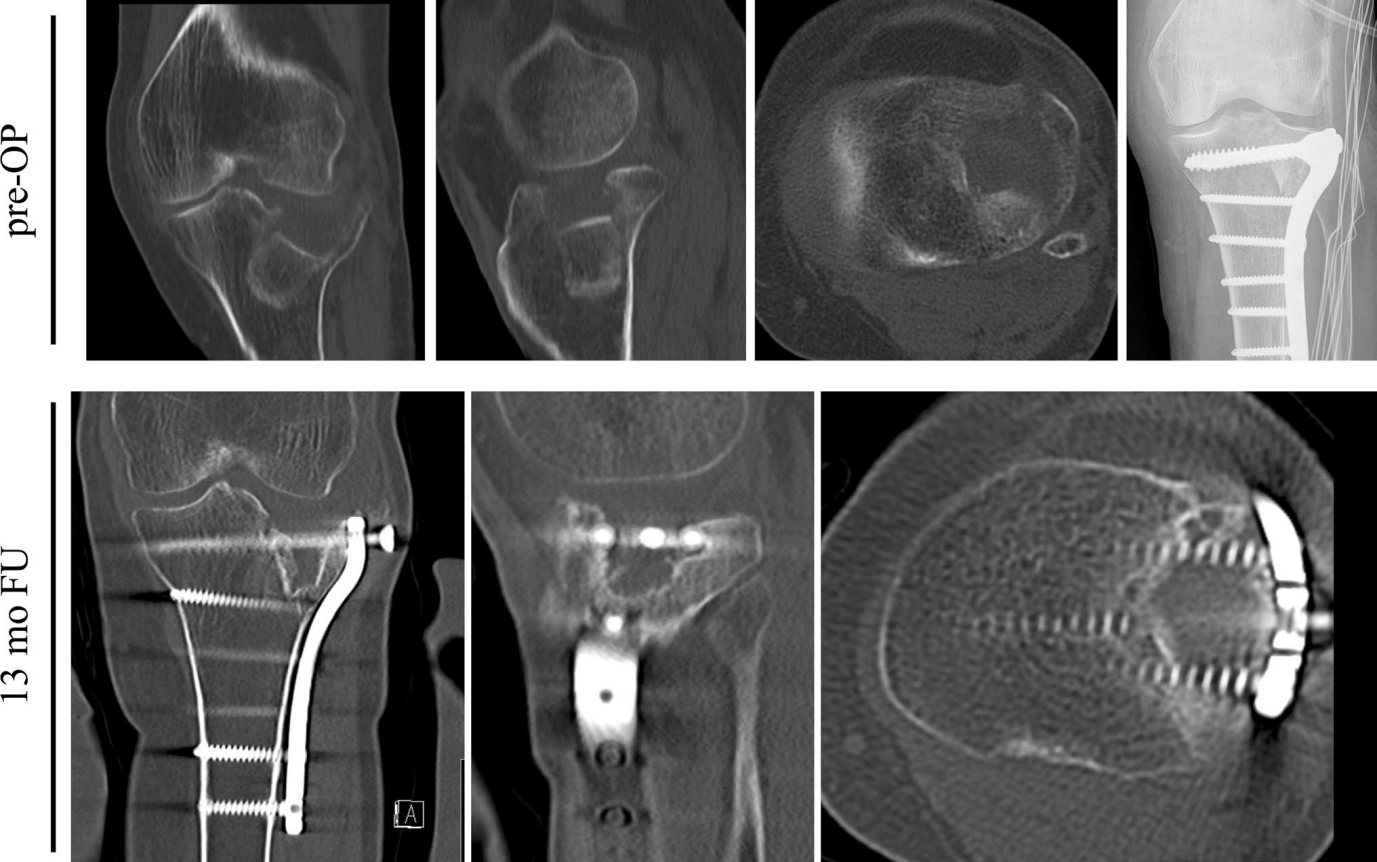
A 78-year-old female with a lateral depression fracture (AO 41-type B2, involving antero-latero-lateral, antero-latero-central segments, *pre-OP*) after a trip and fall. Surgical treatment included an anatomic reduction, transplantation with an autogenic cortico-cancellous iliac crest graft, and fixation with an anterolateral plate. A computed tomography scan at the 13-month follow-up revealed a severe osteochondral defect with a complete dissolution of the autogenic graft. The patient demonstrated a severe vitamin D deficiency of 3.4 ng/mL and persistent hypocalcemia

Analyzing the clinically fair and poor cases (*n* = 6) individually resulted in specific findings (Table 6). In all patients, a secondary loss of fixation could be observed due to severe subchondral bone loss and most probably a concomitant potential IBM. Leading comorbidities included HIV with anti-retroviral therapy (ART) (including abacavir, lamivudine, and nevirapine) and a long-term proton-pump inhibitor (PPI) prescription (Fig. 1), chronic hypocalcemia, chronic alcohol abuse, and long-term smoking (Fig. 2), severe vitamin D deficiency (Fig. 3), severe osteoporosis defined as a DXA T-score of lower than − 2.5 SD and at least two fragility fractures, as well as an unspecified collagenosis with chronic type-c gastritis, subsequent hypocalcemia, and myasthenia gravis.

**Table 6:** Individual failure analysis of clinically poor/fair outcome (Rasmussen)

Patient	AO	Rasmussen (rad/clin)	Specific outcome	Patient-related risk factors	Surgery-related risk factors
#1 (**♂**, 50y, 16 m FU)	C3	14/18	- Loss of reduction PLL, PLC, PMM - Subchondral substance defect	- HIV infection - ART Therapy	- Missing subchondral Jail-screw - No posteromedial buttress
#2 (**♂**, 50y, 14 m FU)	C3	10/14	- Loss of reduction PMM, PMC - Pseudarthrosis	- Hypocalcemia - Smoking - Alcohol abuse	- Screw placement into fracture gap - no posteromedial buttress
#3 (**♀**, 78y, 24 m FU)	B2	12/18	- Subchondral substance defect with loss of reduction AMC, AMM	- Hypocalcemia - Vitamin D deficiency (3.4 ng/mL) - COPD with inhaled corticosteroids	–
#4 left (**♀**, 62y, 30 m FU)	B3	–/19	- Severe pain despite full range of motion with secondary loss of reduction AMC, AMM	- Unspecific collagenosis - Hypocalcemia - Chronic type-c gastritis - Myasthenia gravis	–
#4 right (**♀**, 62y, 30 m FU)	B3	–/19	- Severe pain despite full range of motion with secondary loss of reduction AMC, AMM	- Unspecific collagenosis - Hypocalcemia - Chronic type-c gastritis - Myasthenia gravis	–
#6 (**♂**, 44y, 89 m FU)	C3	14/18	- Severe pain due to lateral knee osteoarthritis - Loss of reduction PLL, PLC	–	- Insufficient articular reconstruction of the postero-latero-central and postero-latero-lateral segments

*FU* follow-up, segments according to ten-segment classification: *AMM* antero-medio-medial, *AMC* antero-medio-central, *PMM* postero-medio-medial, *PMC* postero-medio-central, *PLL* postero-latero-central, *PLC* postero-latero-lateral, *HIV* humane immune insufficiency, *ART* anti-retroviral therapy, *COPD* chronic obstructive pulmonary disease, *FU* follow-up, *m* months

## Discussion

The present study demonstrated a good-to-excellent clinical outcome in more than 95% of the cases [1, 27–30]. Specifically, 41-type B fractures showed a better outcome than that of 41-type C fractures. We found that the presence of potential IBM due to influencing comorbidities or medication was an independent risk factor for a poor or fair clinical outcome, especially in 41-type B fractures. In accordance with the previous studies, a great number of patients with severe bony destruction reported a good-to-excellent clinical outcome despite postoperative radiological peculiarities [8, 14, 16, 36, 37] as well as an even better outcome with unilateral plateau fracture [10, 15, 38].

Common comorbidities, such as type II diabetes or arterial hypertension, have not been associated with clinical outcomes [6, 7, 10]. However, reports analyzing the risk factors of bone metabolism in a cohort of TPF are rare. In the current study, all but one patient with a fair-to-poor clinical outcome demonstrated at least one risk factor for IBM and consequent fracture healing (Table 5). One patient showed secondary loss of reduction in the posterolateral and posteromedial segments, and that patient presented with HIV and ART with abacavir, lamivudine, and nevirapine. HIV itself is a risk factor for impaired fracture healing [39]. Treated patients with HIV have a 1.98-to-3.69 times higher fracture rate compared to healthy controls [40]. In addition, ART regimens induce increased bone loss [41]. However, modern regimens with abacavir–lamivudine and nevirapine have demonstrated fewer decreases in bone mineral density and fewer increases in bone turnover [42]. In addition, the patient was exposed to long-term PPI therapy; this causes iatrogenic hypochlorhydria, which itself is associated with decreased bone mass, decreased bone mineralization, and consequently increased fracture risk [43–45]. PPI-induced hypochlorhydria was also detected in another patient with bilateral fractures and secondary loss of reduction. She presented with chronic type-c gastritis, a subsequent chronic hypocalcemia, and unknown collagenosis, which were responsible for a number of insufficiency fractures in the patient’s history. She also suffered from myasthenia gravis, which is associated with significant bone loss independent of corticosteroid use [46]. Common risk factors for delayed fracture healing or fragility fractures, such as chronic alcohol abuse and severe smoking, were not statistically associated with impaired clinical outcomes in the present study, but they may have been one of the major triggers in one patient with secondary loss of reduction and a substantial subchondral bone loss (Fig. 2) [47]. Additionally, vitamin D deficiency was commonly observed, and even though its role in fracture healing is still under debate, it constitutes one of the most important determinants of skeletal health [47, 48].

Numerous studies that have analyzed the outcome of TPF have identified several risk factors for poor clinical and radiological outcome, such as fracture complexity, leg axis malreduction [9], involvement of the posterior column [7], residual articular depression of more than 2 mm [9, 13], or postoperative malreduction due to an impaired intraoperative fragment visualization [26, 49], while the impact of associated soft-tissue injuries is still under debate [50, 51]. In the individual failure analysis of the present study, two of six cases may be explained solely by a potential IBM. The other four fractures showed secondary loss of reduction. While risk factors for a potential IBM were found in all cases, surgical considerations should also be included. Two cases involved the posterior column with specific involvement of the PLL, PLC, and both posteromedial segments (posteromedial shear fragments) without a supporting posteromedial buttress plate, a direct subchondral screw placement with the strongest bone stock in the tibial plateau, or malreduced posterolateral fragments due to an insufficient initial surgical approach. Addressing the posterior column has been identified as an important prognostic factor with respect to functional outcomes [6, 7].

The most important objective clinical limitation, even in patients with good-to-excellent clinical outcome and especially in 41-type C fractures, was impaired ROM. All patients were encouraged to achieve full ROM within three months after surgery through strict rehabilitation protocols. ROM may still be improved up until the sixth month after surgery, but it may be impaired afterwards if it is not fully accomplished by the sixth month [52]. The most important risk factor is time spent on the external fixator and bicondylar fracture involvement, which is supported by the present results [53]. Hence, limited ROM in TPF is common and should be the focus of postoperative rehabilitation [53, 54].

This study has some limitations. Due to its retrospective nature, data acquisition was based on patient records without a standardized assessment of systemic bone mineral disorders, including dual-energy X-ray absorptiometry or serum analyses of 25-OH vitamin D, phosphate, or creatinine levels. All medical records were carefully screened, with a special emphasis on calcium levels and secondary indicators for potential IBM. Additionally, given the complexity of different fracture patterns, the methods of ORIF differed among the fracture types, resulting in treatment bias [5, 37]. However, this limitation reflects everyday clinical practice in orthopedic trauma surgery [15]. In addition, postoperative CT scans were performed in only 28 patients. However, radiological evaluation to assess the Rasmussen score is established on plain radiographs [33, 55].

## Conclusion

In this multicenter retrospective cohort study, the mid-to-long-term clinical outcome after ORIF of TPF was good-to-excellent in the vast majority of the cases. Potential IBM was an independent risk factor for a poor-to-fair clinical outcome. The most important functional limitation was impaired ROM.
